# Antifatigue Potential Activity of *Sarcodon imbricatus* in Acute Excise-Treated and Chronic Fatigue Syndrome in Mice via Regulation of Nrf2-Mediated Oxidative Stress

**DOI:** 10.1155/2018/9140896

**Published:** 2018-06-28

**Authors:** Xue Wang, Yidi Qu, Yongfeng Zhang, Shaopeng Li, Yiyang Sun, Zepeng Chen, Lirong Teng, Di Wang

**Affiliations:** ^1^School of Life Sciences, Jilin University, Changchun 130012, China; ^2^Zhuhai College of Jilin University, Jilin University, Zhuhai 519041, China

## Abstract

*Sarcodon imbricatus* (SI), a precious edible fungus, contains 35.22% of total sugar, 18.33% of total protein, 24 types of fatty acid, 16 types of amino acid, and 8 types of minerals. Encouragingly, it is rich in potential antioxidants such as total polyphenols (0.41%), total sterols (3.16%), and vitamins (0.44%). In the present study, the antifatigue properties of SI and its potential mechanisms of action were explored by the experiments on acute excise-treated mice and chronic fatigue syndrome (CFS) mice. SI (0.25, 0.5, and 1 g/kg) significantly enhanced exercise tolerance in the weight-loaded forced swimming test (FST) and rota-rod test (RRT) and reduced the immobility in the tail suspension test on CFS mice. SI markedly increased the levels of glycogen in the liver and adenosine triphosphate (ATP) in the liver and muscle and decreased the lactic acid (LD) and blood urea nitrogen (BUN) content in both acute swimming-treated mice and CFS mice. SI improved the endogenous cellular antioxidant enzyme contents in the two mouse models by improving the activities of superoxide dismutase (SOD) and glutathione peroxidase (GSH-Px) and reducing reactive oxygen species (ROS) and malondialdehyde (MDA) levels in serum, liver, and muscle, respectively. In CFS mice, the enhanced expression levels of nuclear factor erythroid-2-related factor 2 (Nrf2), SOD1, SOD2, heme oxygenase-1 (HO-1), and catalase (CAT) in the liver were observed after a 32-day SI administration. Our data indicated that SI possessed antifatigue activity, which may be related to its ability to normalize energy metabolism and Nrf2-mediated oxidative stress. Consequently, SI can be expected to serve as a novel natural antifatigue supplement in health foods.

## 1. Introduction

Fatigue, caused by fierce stress from physical and mental work, is a decreased performance under subhealthy conditions [1]. Fast-paced lifestyles, intense competitive pressures, and irregular eating and drinking habits put people at risk of fatigue. If fatigue symptoms cannot be alleviated in time, patients will experience chronic fatigue syndrome (CFS), which is defined as the persistent or recurrent severe fatigue (more than 6 months) accompanied by apathetic, tender lymphadenopathy, body aches, headaches, unrefreshing sleep, inattention, and lower work efficiency [2]. CFS increases the risk of neuropsychiatric problems, such as depression and anxiety [3]. Depression mood is recognized as an important characteristic indicator within CFS patients [4].

Oxidative stress, a well-characterized factor, has received widespread attention as a bridge between fatigue and CFS. Oxidative stress is triggered by the overproduction of reactive oxygen species (ROS), and it attacks large molecules and cell organs [5] which leads to an injured body. Increased oxidative stress and decreased antioxidant defenses are positively correlated with the severity of symptoms in CFS [6]. Free radicals are regarded as an important indicator of impairment of skeletal muscle function, and intense exercise induces excessive ROS production. Lipid peroxidation caused by ROS leads to structural damage and cell or organelle dysfunction [7]. According to previous literature reports, skeletal muscle and liver mitochondria were susceptible to lipid peroxidation-induced damage during exercise [8]. And prolonged oxidative stress will trigger CFS [9]. Nuclear factor-erythroid 2-related factor 2/antioxidant responsive element (Nrf2/ARE) is one of the most important defense mechanisms of the body's cells against oxidative damage [10].

Till now, the etiology of CFS is still not clear. Although some medicines including immunostimulants, immunosuppressants, antidepressants, hypnotics, analgesics, and antihistamines were used for the treatments to CFS-related diseases, no satisfactory effects were obtained in clinics due to no optimistic long-term efficacy and various side effects [11, 12]. Since nutrient supplementation positively enhances exercise capacity, researches attempt to seek a safe and effective anti-CFS agent from natural products, which people can take as the “tonics.” Recently, China has carried out preclinical studies and clinical trials of CFS, with special emphasis on the use of traditional Chinese herbal medicine [13, 14]. Fungus, containing various nutritional ingredients, exhibits multiple activities such as antioxidation and antifatigue [15, 16]. Antioxidant active ingredients in natural medicines mainly include polysaccharides, polyphenols, tetraterpenes, sterols, and vitamins, which realize antioxidant activity by scavenging free radicals, terminating the progress of chain oxidation, and improving the antioxidant capacity of the body [17]. *Sarcodon imbricatus* (SI), belonging to the family of *Basidiomycotina* and *Aphyllophorales*, is an edible and medicinal fungus, widely distributed in Central Europe and in North America [18] and also produced in Tibet, Gansu, Anhui, northwestern Yunnan, and western Sichuan of China [19]. Although SI is described to show various pharmacological activities including anti-inflammation and anticancer in the folk, previous studies mainly focused on its chemical component analysis and polysaccharide isolation [20, 21]. Our group has already confirmed the immunomodulatory property of *S. imbricatus* water extracts in the cyclophosphamide- (CTX-) induced immunosupressive mouse model, which is related to its modulation on oxidative stress [22]. Encouragingly, based on these data, we speculated that SI has certain effects on improving exercise endurance and relieving fatigue due to its oxidation resistance.

For this purpose, in the present study, we analyzed the components of SI systematically first and then investigated its antifatigue effect properties and potential mechanisms in acute excise-treated and CFS mouse models. Valuable and useful information about the bioactivity of SI as a functional food supplement will be provided in our data.

## 2. Materials and Methods

### 2.1. Plant Material and Preparation

SI were collected from the broad-leaved forest area of Yunnan in September 2015, which are taxonomically identified by the Engineering Research Center of Chinese Ministry of Education for Edible and Medicinal Fungi, Jilin Agricultural University, Changchun, China. Dried SI were pulverized into powder by a flour mill and sieved through an 80-mesh sieve. It was dark brown and stored in a desiccator for subsequent experiments.

### 2.2. Measurement of the SI Components

#### 2.2.1. Main Components

The main nutrition and quality components of the SI fruiting body were systematically determined according to the previous studies and national standards. The Folin-Ciocalteu method [23], UV spectrophotometric assay [24, 25], HPLC methods [26], phenol-sulfuric acid determination [27], 3,5-dinitrosalicylic acid colorimetric estimation [28], vanillin-glacial acetic acid and perchloric acid colorimetric spectrophotometry [29], the aluminium chloride colorimetric method [30], the periodate oxidation method [31], the petroleum benzine extraction method [32], the ashing method [33], and the Kjeldahl method [34] were used to analyze the levels of polyphenols, total content of carotenoids and sterols, vitamins, total sugar, reducing sugar, triterpenoids, flavonoids, mannitol, crude fat, total ash, and total protein, respectively.

#### 2.2.2. Amino Acid Analysis

SI was hydrolyzed using 6 mol/L of HCl at 110°C for 24 h. After vacuum drying, the samples were dissolved in 1 mL of a buffer with pH 2.2. A quantitative analysis of the amino acids was carried out using an automatic amino acid analyzer (L-8900, Hitachi, Japan).

#### 2.2.3. Minerals

Minerals of SI were carried out according to previous studies with some modifications [35]. Briefly, SI (0.5 g of each time) was placed in a porcelain citrus pot and completely ashed and then dissolved in nitric acid (5 mL). The digestion procedure was set as follows: raising the room temperature to 120°C, 0–5 min; holding at 120°C, 6–7 min; raising from 120 to 180°C, 8–17 min; and holding at 180°C, 18–32 min. After cooling at room temperature, the solution was transferred into a 50 mL volumetric flask and diluted to 50 mL with deionized water. Subsequently, the levels of potassium (K), sodium (Na), calcium (Ca), iron (Fe), zinc (Zn), manganese (Mn), copper (Cu), selenium (Se), mercury (Hg), arsenic (As), cadmium (Cd), chromium (Cr), and lead (Pb) were detected by inductively coupled plasma-atomic emission spectrometry (ICP-AES, Thermo Elemental, Franklin, MA).

#### 2.2.4. Fatty Acids

SI was extracted using a ratio of chloroform : methanol 2 : 1 (*v* : *v*), evaporated under the conditions of 80°C, and then mixed with potassium hydroxide-methanol solution (4 g potassium hydroxide : 100 mL methanol) at 50°C for 10 min. 1 mL of 20% BF_3_ solution was added to the samples, and then the samples were incubated at 50°C for another 15 min. Finally, the samples were mixed with hexane. The hexane layer was washed with water until neutral, and the levels of fatty acids were analyzed using a gas chromatography-mass spectrometer (QP2010, Shimadzu, Japan).

### 2.3. Animal Care and Experimental Procedure

Experimental protocol was approved by the Institution Animal Ethics Committee of Jilin University (20160208). One hundred and ten Kunming male mice (4–6 weeks, 18–22 g, specific pathogen-free (SPF) grade) (SCXK (JI)-2017-0001) purchased from the lab animal center of Jilin University were housed under a controlled environment at a temperature of 22 ± 2°C and moderate humidity of 50 ± 10% with a 12/12 h light/dark cycle and fed an autoclaved standard chow and water *ad libitum*. Mice were acclimatized for one week and then were used in the following experiments.

#### 2.3.1. Acute Excise-Treated Mouse Model Establishment and Agent Treatment Procedure

**Figure 1:**
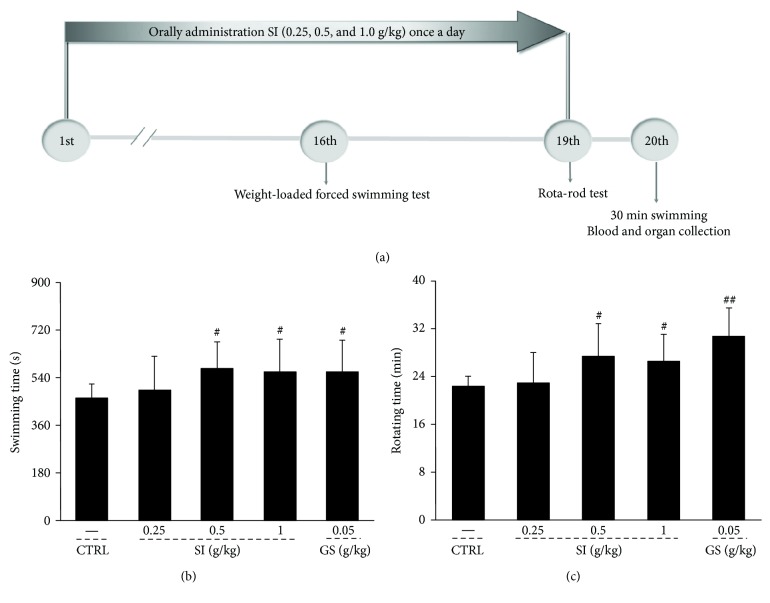
(a) The experimental protocol and drug administration procedure on acute exercise-treated mice. The effects of SI and GS on (b) weight-loaded forced swimming test and (c) rota-rod test in normal mice. Data were analyzed using a one-way ANOVA followed by Dunn's test and expressed as means ± SD (*n* = 10). ^#^*P* < 0.05 and ^##^*P* < 0.01 versus the control group. SI: *S. imbricatus*; GS: *Ginsenoside*.

Fifty mice were taken out randomly according to body weight and divided into five groups (*n* = 10/group) and orally treated with 0.5% of sodium carboxymethyl cellulose (CMC-Na, 0.2 mL/20 g) (control mice), 0.05 g/kg of ginsenoside (GS, 0.2 mL/20 g) dissolved in 0.5% of CMC-Na (positive control mice), and SI at doses of 0.25 g/kg (0.2 mL/20 g), 0.5 g/kg (0.2 mL/20 g), and 1.0 g/kg (0.2 mL/20 g) dissolved in 0.5% of CMC-Na once per day for 18 days. Oral gavage treatment was performed at 9 : 00 every day. At the 16th day and the 19th day, after SI administration for 30 min, the weight-loaded forced swimming test (FST) and rota-rod test (RRT) were performed to evaluate the endurance capacity of mice in each group, respectively. At the 20th day, all mice were forced to swim for 30 min without loads, and then blood was sampled from the caudal veins. After sacrificing, liver and muscle were collected from each mouse rapidly. The detailed experimental protocol and drug administration are shown in Figure 1(a).

#### 2.3.2. CFS Mouse Model Establishment and Agent Treatment Procedure

**Figure 2:**
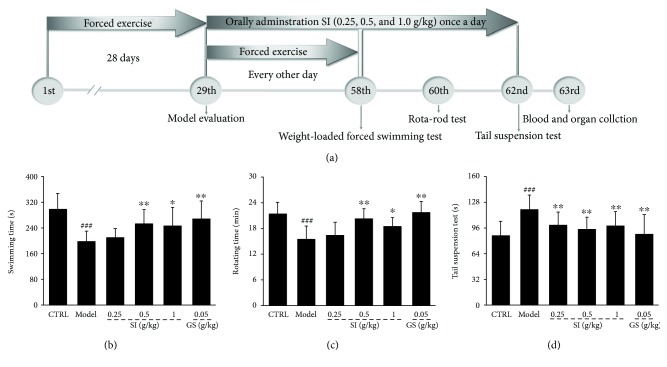
(a) The experimental protocol and drug administration procedure on CFS mice. The effects of SI and GS on (b) weight-loaded forced swimming test, (c) rota-rod test, and (d) tail suspension test in CFS mice. Data were analyzed using a one-way ANOVA followed by Dunn's test and expressed as means ± SD (*n* = 10). ^###^*P* < 0.001 versus the control group; ^∗^*P* < 0.05 and ^∗∗^*P* < 0.01 versus the model group. SI: *S. imbricatus*; GS: *Ginsenoside*.

Based on previous reports [36, 37], fifty mice were exposed to different stimuli including cold water swimming (15°C ± 1°C) for 10 min, exhaustive running 15 min, rota-rod for 15 min, and sleep deprivation once per day for 4 weeks. The same stressor was not applied continuously for two days. Another 10 mice receiving no stimuli for 4 weeks serve as the control group. FST was applied to test whether the CFS mice were established successfully. At the 29th day, CFS mice were divided into five groups randomly (*n* = 10/group) and orally administrated with 0.5% of CMC-Na (0.2 mL/20 g) (model mice), 0.05 g/kg of GS (0.2 mL/20 g) dissolved in 0.5% of CMC-Na (positive control mice), and SI at doses of 0.25 g/kg (0.2 mL/20 g), 0.5 g/kg (0.2 mL/20 g), and 1.0 g/kg (0.2 mL/20 g) dissolved in 0.5% of CMC-Na once per day for 32 days. Mice received different stimuli from the 29th day to the 57th day every other day. The control group mice were orally treated with 0.5% of CMC-Na (0.2 mL/20 g) for 32 days. At the 58th day, 60th day, and 62nd day, FST, RRT, and tail suspension test (TST) were performed, respectively. At the 63rd day, blood was sampled from the caudal veins. After sacrificing, the liver and muscle were collected from each mouse rapidly. The detailed experimental protocol and drug administration are shown in Figure 2(a).

### 2.4. Animal Behavioral Tests

#### 2.4.1. Weight-Loaded Forced Swimming Test

Mice were placed individually in a swimming pool (height: 30 cm, diameter: 25 cm) at 25°C ± 1°C, in which the mice could swim freely but were prohibited to touch the bottom. Lead blocks of 10% body weight were loaded on the tail root of each mouse. The mice were assessed to be exhausted when they failed to rise to the surface of water to breathe within a period of 8 s. Their exhaustive swimming time was recorded.

#### 2.4.2. Rota-Rod Test

Mice were, respectively, placed on a rota-rod (ZB-200, Chengdu Taimeng Science Technology Co., Ltd., Chengdu, China) at 15 rpm for training three times. In the formal test, mice were placed on the rota-rod at 15 rpm, respectively, until they were exhausted and dropped from the rod. The total running time was recorded.

#### 2.4.3. Tail Suspension Test

The tail suspension time not only reflects the state emotion of the animal's psychological endurance but also displays the animal's physical endurance. Mice were, respectively, suspended 1 m above the floor using an adhesive tape, positioned about 1 cm from the tip of the tail. The total duration of immobility, which can be defined as motionless hanging without any struggling movements, was recorded during the last 4 min of the whole 6 min test period.

### 2.5. Sample Preparations and Analysis of Biochemical Parameters

Serum was isolated by centrifugation at 4000 rpm for 15 min at room temperature. One part of the liver and muscle was homogenized to 10% solution with normal saline at 4°C. The levels of blood urea nitrogen (BUN) (C013-2), lactic acid (LD) (A019-2), adenosine triphosphate (ATP) (A003-1), glycogen (A043) (Nanjing Jiancheng Biological Company, Nanjing, China), reactive oxygen species (ROS) (CK-E91516), superoxide dismutase (SOD) (CK-E20348), malondialdehyde (MDA) (CK-E20347), and glutathione peroxidase (GSH-Px) (CK-E92669) (Shanghai Yuanye Bio-Technology Co., Ltd., Shanghai, China) in serum, liver, and muscle were detected by ELISA method according to the manufacturer's instructions.

### 2.6. Western Blot Analysis

One part of liver tissues obtained from CFS mice was extracted with lysis buffer (RIPA with protease and phosphatase inhibitor) for 30 min on ice and then centrifuged at 10000 rpm for 10 min at 4°C to remove the precipitate. The concentration of total protein was determined by a bicinchoninic acid (BCA) protein assay kit (Merck Millipore, USA). An equal amount of denatured protein samples (40 *μ*g) was loaded per well for 12% SDS-polyacrylamide gel electrophoresis (Bio-Rad, USA) and transferred to PVDF membranes. The membranes were blocked using 5% bovine serum albumin (BSA) at room temperature for 2 h. The blots were incubated with the appropriate concentration of specific antibody overnight at 4°C. Primary antibodies Nrf2 (ab137550), SOD1 (ab16831), SOD2 (ab131443), heme oxygenase-1 (HO-1) (ab25901), catalase (CAT) (ab7970) (Abcam, Cambridge, USA), and glyceraldehyde 3-phosphate dehydrogenase (GAPDH) (ABS16) (Merck Millipore, Darmstadt, Germany) were diluted at 1 : 2000. The bonds were washed with TBS buffer plus 0.1% Tween-20 for five times and then incubated with horseradish peroxidase-conjugated goat anti-rabbit secondary antibody (sc-3836) (Santa Cruz Biotechnology, Santa Cruz, USA) for 4 h at 4°C. The bands were established and fixed by an ECL Advance kit. The quantification of protein expression was determined using the ImageJ 1.46 software (Rasband, Bethesda, MD, USA).

### 2.7. Statistical Analysis

The data were analyzed using SPSS 16.0 software (IBM Corporation, USA). The results were presented as means ± standard deviation (SD), and the statistical significance of each difference was determined using a one-way analysis of variance (ANOVA) followed by Dunn's test. In the analysis results, *P* < 0.05 was considered to indicate significant differences.

## 3. Results

### 3.1. Composition of SI

For general nutrition, the SI fruiting body contains 35.22% of total sugar, 3.41% of reducing sugar, 0.04% of triterpenoids, 0.02% of flavonoids, 9.40% of mannitol, 3.02% of crude fat, 9.30% of total ash, 18.33% of total protein, 0.41% of total polyphenols, 3.16% of total sterols (Table 1), and 0.44% of vitamins (Table 2). Among 35 types of fatty acid detected, 24 types of them were found in the SI fruiting body (Table 3). Automatic amino acid analysis showed that the SI fruiting body consists 16 kinds of amino acids, including essential amino acids. Among them, 1.11% of aspartic acid (Asp), 3.04% of glutamic acid (Glu), and 1.10% of alanine (Ala) were noted, which shows higher levels than do other amino acids (Table 4). For mineral elements, the SI fruiting body contains K (3957.0 mg/100 g), Fe (78.4 mg/100 g), Ca (68.0 mg/100 g), Zn (10.9 mg/100 g), Na (14.5 mg/100 g), Mn (3.3 mg/100 g), and Cu (3.3 mg/100 g) (Table 5).

### 3.2. SI-Enhancing Exercise Capacities of Normal Mice and CFS Mice

The animal behavioral experiment intuitively reflects the antifatigue properties of test agents [38]. SI showed similar enhancing effects on exercise endurance of normal mice and CFS mice as that of GS. In normal mice, SI improved the exhaustion swimming time > 20.9% compared with control mice in FST (*P* < 0.05; Figure 1(b)). 0.5 g/kg and 1.0 g/kg of SI prolonged the excise time up to 18.7% and 19.3% compared with the control mice in RRT (*P* < 0.05; Figure 1(c)). In CFS mice, compared with the control group, the reduced exercise time was observed in FST (*P* < 0.001; Figure 2(b)) and RRT (*P* < 0.001; Figure 2(c)), and the increased immobility duration was found in TST (*P* < 0.001; Figure 2(d)). After 32-day oral administration of SI, compared with CFS mice, SI resulted in >24.3% and > 19.1% enhancements on exercise time in FST (*P* < 0.05; Figure 2(b)) and RRT (*P* < 0.05; Figure 2(c)), and a >21.2% reduction on immobility duration time in TST (*P* < 0.01; Figure 2(d)).

### 3.3. Effects of SI on the Levels of BUN, LD, ATP, and Glycogen in Serum and Organs of Acute Excise-Treated Mice and CFS Mice

**Figure 3:**
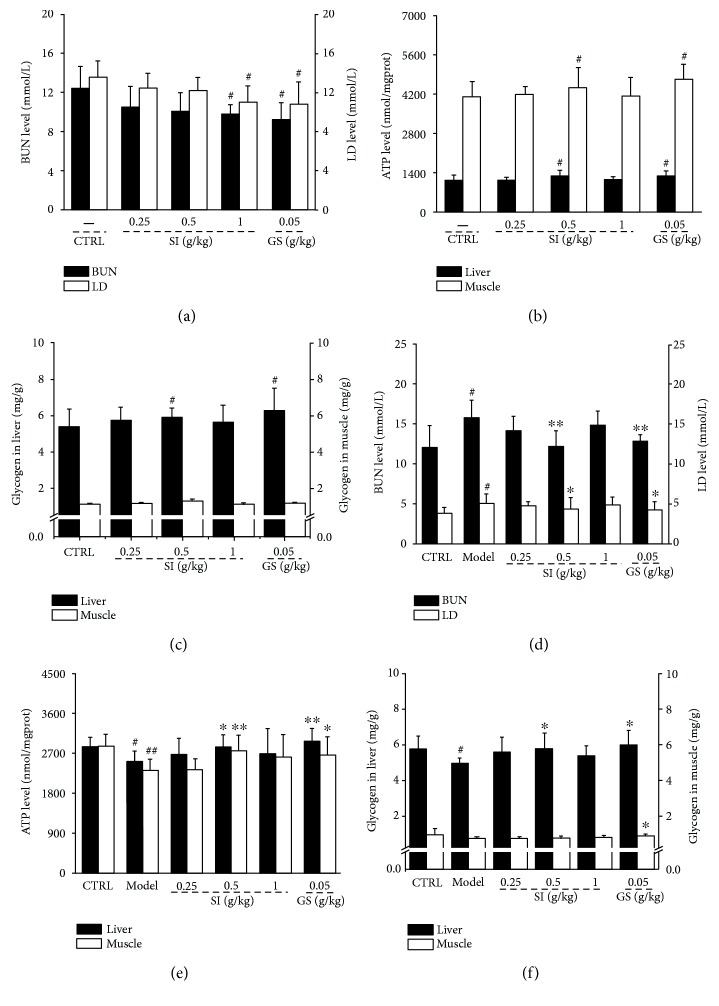
Mice were treated with SI (0.25, 0.5, and 1.0 g/kg) and GS (0.05 g/kg) for 18 days. After a 30 min swimming, the levels of (a) BUN and LD in serum, (b) ATP, and (c) glycogen in the liver and muscle were detected via ELISA kit. CFS mice were treated with SI (0.25, 0.5, and 1.0 g/kg) and GS (0.05 g/kg) for 32 days. The levels of (d) BUN and LD in serum, (e) ATP, and (f) glycogen in the liver and muscle were detected via ELISA kit. Data were analyzed using a one-way ANOVA followed by Dunn's test and expressed as means ± SD (*n* = 10). ^#^*P* < 0.05 and ^##^*P* < 0.01 versus the control group; ^∗^*P* < 0.05 and ^∗∗^*P* < 0.01 versus the model group. SI: *S. imbricatus*; GS: *Ginsenoside*.

In acute excise-treated mice, the 18-day SI treatment resulted in 21.2% and 18.6% reduction on the BUN and LD in serum compared to control mice (*P* < 0.05; Figure 3(a)). SI at a dose of 0.5 g/kg enhanced the ATP levels by 13.6% and 8.0% in the liver and muscle of acute excise-treated mice (*P* < 0.05; Figure 3(b)). Furthermore, compared with the control group, SI only significantly enhanced the glycogen levels in the liver (*P* < 0.05; Figure 3(c)), but not in the muscle (*P* > 0.05; Figure 3(c)) in acute excise-treated mice.

In CFS mice, compared with the control group, the enhanced levels of BUN (*P* < 0.05; Figure 3(d)) and LD (*P* < 0.05; Figure 3(d)) in serum and the decreased levels of hepatic glycogen (*P* < 0.05; Figure 3(f)) and ATP in the liver (*P* < 0.05; Figure 3(e)) and muscle (*P* < 0.01; Figure 3(e)) were noted. SI displayed similar effects as that of GS except for those on muscle glycogen levels, which were only enhanced after GS administration (*P* < 0.05; Figure 3(f)). Compared with nontreated CFS mice, SI treatment resulted in 23.0% and 15.4% decrement on serum levels of BUN (*P* < 0.01; Figure 3(d)) and LD (*P* < 0.05; Figure 3(d)), respectively. Moreover, SI increased the ATP levels by 12.4% and 19.0% in the liver (*P* < 0.05; Figure 3(e)) and muscle (*P* < 0.05; Figure 3(e)) of CFS mice. SI only showed beneficial effects on hepatic glycogen levels, which were enhanced by 17.4% in SI-treated CFS mice (*P* < 0.05; Figure 3(f)).

### 3.4. Effects of SI on the Levels of Oxidative Stress Factors in Acute Excise-Treated Mice and CFS Mice

Excessive ROS destroys the balance between oxidation and antioxidation, resulting in the occurrence of oxidative stress [39]. MDA, a polyunsaturated fatty acid peroxide degradation product, indirectly reflects the degree of cellular attack and damage by free radicals. SOD is against the damage from oxygen free radicals; meanwhile, GSH-Px helps lipid peroxides be catalyzed by reduced glutathione (GSH) [40, 41]. SI showed similar regulatory effects on the levels of oxidative stress-related factors in acute excise-treated mice (Table 6) and CFS mice (Table 7) as that of GS. Compared with the control group, 18-day SI oral administration strongly reduced the levels of ROS (*P* < 0.05) and MDA (*P* < 0.05) and enhanced the levels of SOD (*P* < 0.05) and GSH-Px (*P* < 0.05) in serum and liver of 30 min swimming-treated mice (Table 6). In muscle, SI only reduced the ROS levels and enhanced the SOD concentration (*P* < 0.05), but failed to significantly influence the levels of MDA and GSH-Px (*P* > 0.05; Table 6).

CFS model establishment procedures resulted in levels of MDA and ROS increasing strongly and SOD and GSH-Px reducing in serum, liver, and muscle (*P* < 0.05; Table 7). After the 32-day gavage treatment, SI at doses of 0.5 and 1.0 g/kg decreased the serum, liver, and muscle levels of MDA and ROS back to the normal horizon (*P* < 0.05). Furthermore, SI resulted in 19.4–48.0% enhancement on SOD and 13.2–53.4% enhancement on GSH-Px levels in serum, liver, and muscle compared with the model group (*P* < 0.05).

### 3.5. The Regulatory Effects of SI on Nrf2 Signaling in the Liver of CFS Mice

**Figure 4:**
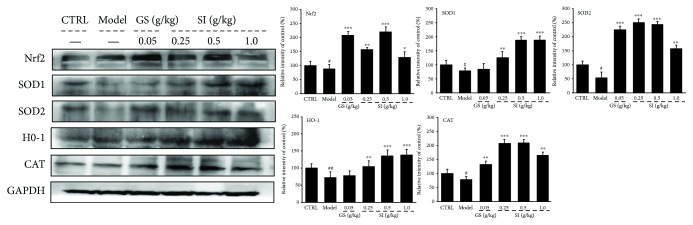
CFS mice were treated with SI (0.25, 0.5, and 1.0 g/kg) and GS (0.05 g/kg) for 32 days. The expression levels of Nrf2, SOD1, SOD2, HO-1, and CAT in the liver were detected via Western blot. Quantification data were normalized by related GAPDH and expressed as means ± SD (*n* = 10). ^#^*P* < 0.05 and ^##^*P* < 0.01 versus the control group. ^∗^*P* < 0.05, ^∗∗^*P* < 0.01, and ^∗∗∗^*P* < 0.001 versus the model group. SI: *S. imbricatus*; GS: *Ginsenoside*.

In order to further reveal the potential mechanisms of antifatigue activities of SI in CFS mice, the expression levels of Nrf2, SOD1, SOD2, HO-1, and CAT in the liver were detected via Western blot. Nrf2 combined with ARE regions of antioxidant enzyme genes and activated these genes for transcription. The levels of Nrf2, SOD1, SOD2, HO-1, and CAT were remarkably downregulated in CFS mice compared with the control group (*P* < 0.05; Figure 4). Compared with the CFS model group, 32-day SI treatment strongly upregulated the expression of Nrf2 and the content of four antioxidant enzymes in the liver (*P* < 0.01; Figure 4). All results indicated that the Nrf2/HO-1 signal pathway can be activated by SI in the tested concentration range.

## 4. Discussion

Fatigue is a common physiological phenomenon and also accompanies with various diseases [42]. In the present study, a comprehensive and systematic experiment was performed to investigate the antifatigue activities of SI and the underlying mechanisms related to oxidative stress in acute excise-treated mice and CFS mice. SI is rich in polysaccharides, proteins, amino acids, and potential antioxidants such as polyphenols, sterols, and vitamins. Both polysaccharides and amino acids have been reported to improve the exercise capability, especially the amino acids, which can markedly retard the catabolism of protein in the muscle during exercise [43–45]. Gly, Pro, and Arg presented from the porcine placenta extract improve glycogen content and CAT and SOD activities and lower the blood levels of LD and alanine aminotransferase [46]. Polyphenolic compounds are involved in neutralizing free radicals, modulating the enzymatic activity, and decomposing peroxide mechanisms to produce antioxidant activities [47]. Sterols are reported to be of great benefit to human health due to their antioxidant activities [48]. Vitamin C enhances the antioxidant capacity of the body mainly by scavenging hydroxyl radicals and cutting off the chain reaction. Meanwhile, vitamin C can work synergistically with vitamin E to exert an antioxidant effect by converting the oxidized form of *α*-tocopherol back to *α*-tocopherol [49]. The antifatigue activity of SI may be related to its rich potential antioxidative nutrient elements. Furthermore, it was confirmed that the heavy metals detected in SI were all within normal limits. In our preliminary experiments, the acute toxicity test showed that SI failed to influence the body weights, water and diet intakes, and organ functions of mice during a 7-day observation. All data reflect the safety of SI in animal experiments.

FST and RRT are used widely to assess the physical strength and the degree of fatigue in animals [50]. The immobility time of the tail suspension test, to a certain extent, can reflect the animal's muscle strength and emotions [37]. The relief from fatigue is the most important factor to improve the exercise endurance. SI significantly prolonged exhaustive swimming time and rata-roding time in normal mice and CFS mice and reduced the immobility time in TST. In order to further confirm the antifatigue activity of SI, biochemical indexes in serum, liver, and/or muscle were also analyzed. SI reduced serum levels of BUN and LD, increased the ATP content in the liver and muscle, and enhanced the concentration of hepatic glycogen. The content of BUN reflects the protein catabolism and the body tolerance to exercise, which serves as a biochemical index to evaluate the degree of fatigue [51]. In the course of vigorous movement, aerobic energy supply changes into anaerobic glycolysis in the muscle, and muscle glycogen is rapidly consumed, which produces a large amount of LD. The buildup of LD in the muscle and blood can cause a decrease in muscle capacity, further leading to exercise-induced fatigue [52]. These alterations were significantly attenuated by SI treatment. LD accumulation can alter the acidic environment in muscle and blood, resulting in overconsumption of phosphate, which hinders ATP synthesis [53]. ATP is the most direct and fastest source of energy. As the energy supplier during ATP production, mitochondria can be damaged by excessive deposition of oxygen free radicals, which will further delay the ATP synthesis [54]. Glycogen, reflecting the body's ability to resist exercise fatigue, can be consumed rapidly under strenuous exercise continuously to provide energy for muscle fiber contraction [55]. All present data confirmed the antifatigue properties of SI.

As reported, acute strenuous exercise and high consumption of energy could accelerate the occurrence of free radicals such as ROS and reactive nitrogen species (RNS) and induce severe oxidative stress bursting [56]. Patients with chronic fatigue syndromes have higher levels of free radicals [57]. Oxidative stress results from the imbalance between oxidant attack, which is due to free radical production, and antioxidant defense, which limits exercise capacity and mitochondrial functions [58]. Overproduced free radicals will attack the fatty acids on the cell membrane and eventually metabolize into MDA, which directly reflect the degree of lipid peroxidation [59]. The accumulation of ROS disturbs the balance of body metabolism and further leads to fatigue symptoms [60]. As important antioxidant enzymes, SOD and GSH-Px are natural scavengers of ROS in bodies [61]. Encouragingly, SI not only regulated the levels of these prooxidant and antioxidant factors in serum, liver, and muscle of acute excise-treated mice and CFS mice but also modulated the expression levels of Nrf2 signaling-related proteins in the liver of CFS mice. Nrf2 is the key regulator of cellular oxidation in the transcriptional level, which directly controls the concentration of SOD, HO-1, and CAT [62]. HO-1 helps to convert heme into biliverdin, which, in turn, is converted into bilirubin, a potent antioxidant [63]. When ROS accumulates excessively, Nrf2 is activated and accumulated in the cytoplasm [64]. Nrf2-deficient mice exhibit extreme vulnerability to oxidative stress in hepatic and gastric tissues [65]. Via improving the activity of Nrf2 in bodies, the oxidative stress damage can be effectively prevented [66]. It has demonstrated in numerous *in vivo* studies that activation of Nrf-2 can counteract oxidative stress and thus reduce fatigue [67]. Generally, the basic intracellular expressions of Nrf-2 are not sufficient to completely suppress oxidative stress. At this point, antioxidant compounds exhibit extraordinary potential to increase the inducible expression of Nrf-2, thereby contributing to a production of large quantities of antioxidants. Altogether, SI shows antifatigue activities in acute exercise-treated mice and CFS mice via regulating Nrf2 signaling-mediated oxidative stress.

In the present study, we only analyzed the antifatigue activities of the SI fruiting body, but not its mycelium obtained by submerged fermentation. The advantages of submerged fermentation have been reported widely such as the shorter growth cycle, stability chemical composition, and controllable biosynthesis processes. Encouragingly, the optimum submerged fermentation conditions for SI mycelium culture have been obtained by the previous study [21]. In our subsequent experiments, the differences in antifatigue activities between the SI fruiting body and SI mycelia will be investigated.

In conclusion, we first demonstrated the antifatigue effects of SI in acute excise-treated mice and CSF mice. SI increased exercise endurance in FST and RRT and reduced the immobility time in TST of CFS mice. SI reduced the levels of BUN and LD, enhanced ATP and glycogen storage, and promoted antioxidant ability by suppressing MDA and ROS levels and increasing SOD and GSH-Px levels. Further data reveal that SI displays the antifatigue ability via regulating Nrf2-mediated oxidative stress. Taken together, our results suggested that SI might be a good candidate for developing a new antifatigue functional food supplement.

## Figures and Tables

**Table 1:** Main components of SI.

Compounds	Contents (%)	Compounds	Contents (%)	Compounds	Contents (%)
Total sugar	35.22	Mannitol	9.40	Polyphenols	0.41
Reducing sugar	3.41	Crude fat	3.02	Carotenoids (×10^−3^)	0.21
Triterpenoids (×10^−2^)	4.12	Total ash	9.30	Sterols	3.16
Flavonoids (×10^−2^)	2.05	Total protein	18.33		

SI: *Sarcodon imbricatus*

**Table 2:** The composition of vitamins in SI.

Compounds	Contents (mg/kg)	Compounds	Contents (mg/kg)	Compounds	Contents (mg/kg)
Vitamin A	0.12	Vitamin B_3_ (×10^3^)	3.16	Vitamin D_2_ (×10^2^)	1.10
Vitamin B_1_	ND^①^	Vitamin B_6_	ND^②^	Vitamin D_3_	ND^③^
Vitamin B_2_	28.68	Vitamin C (×10^3^)	1.06	Vitamin E	ND^④^

SI: *Sarcodon imbricatus*; ND^①^: not detected (the detection limit was 0.54 mg/kg); ND^②^: not detected (the detection limit was 2.92 mg/kg); ND^③^: not detected (the detection limit was 0.08 mg/kg); ND^④^: not detected (the detection limit was 1.32 mg/kg).

**Table 3:** The composition of fatty acids.

Compounds	Contents (‰)	Compounds	Contents (‰)	Compounds	Contents (‰)
Octoic acid (C8:0)	ND^①^	Heptadecenoic acid (C17:1) (×10^−2^)	3.02	Docosanoic acid (C22:0) (×10^−2^)	8.30
Capric acid (C10:0)	ND^②^	Stearic acid (C18:0) (×10^−1^)	4.70	Eicosatrienoic acid (C20:3n6)	ND^⑦^
Undecanoic acid (C11:0) (×10^−2^)	0.51	Trans-oleic acid (C18:1n9t) (×10^−2^)	3.61	Erucic acid (C22:1n9)	ND^⑧^
Lauric acid (C12:0) (×10^−2^)	1.72	Oleic acid (C18:1n9c)	4.89	Eicosatrienoic acid (C20:3n3) (×10^−2^)	1.92
Tridecanoic acid (C13:0) (×10^−2^)	0.40	*trans*-Linoleic acid (C18:2n6t) (×10^−2^)	0.62	Arachidonic acid (C20:4n6)	ND^⑨^
Myristic acid (C14:0)	0.07	Linoleic acid (C18:2n6c)	15.26	Tricosanoic acid (C23:0) (×10^−2^)	1.32
Myristoleic acid (C14:1)	ND^③^	Arachidic acid (C20:0) (×10^−2^)	4.43	Docosadienoic acid (C22:2n6) (×10^−2^)	2.20
Pentadecanoic acid (C15:0)	0.72	*γ*-linolenic acid (C18:3n6)	ND^⑤^	Tetracosanoic acid (C24:0) (×10^−2^)	5.12
Pentadecenoic acid (C15:1)	ND^④^	Eicosanoic acid (C20:1n9)	ND^⑥^	Eicosapentaenoic acid (C20:5n3)	ND^⑩^
Hexadecanoic acid (C16:0)	1.97	*α*-Linolenic acid (C18:3n3) (×10^−2^)	5.34	Nervonic acid (C24:1n9) (×10^−1^)	0.05
Palmitoleic acid (C16:1)	0.07	Heneicosanoic acid (C21:0) (×10^−2^)	0.41	Docosahexaenoic acid (C22:6n3)	ND^⑪^
Heptadecanoic acid (C17:0)	0.07	Eicosadienoic acid (C20:2)	0.04		

ND^①^: not detected (the detection limit was 4.20 mg/kg); ND^②^: not detected (the detection limit was 3.83 mg/kg); ND^③^: not detected (the detection limit was 2.82 mg/kg); ND^④^: not detected (the detection limit was 2.64 mg/kg); ND^⑤^: not detected (the detection limit was 2.51 mg/kg); ND^⑥^: not detected (the detection limit was 4.77 mg/kg); ND^⑦^: not detected (the detection limit was 2.68 mg/kg); ND^⑧^: not detected (the detection limit was 2.42 mg/kg); ND^⑨^: not detected (the detection limit was 4.66 mg/kg); ND^⑩^: not detected (the detection limit was 3.31 mg/kg); ND^⑪^: not detected (the detection limit was 4.33 mg/kg).

**Table 4:** The composition of amino acids in SI.

Compounds	Contents (%)	Compounds	Contents (%)	Compounds	Contents (%)
Aspartic acid (Asp)	1.11	Valine (Val)	0.62	Lysine (Lys)	0.61
L-Threonine (Thr)	0.64	DL-Methionine (Met)	0.40	Histidine (His)	0.10
Serine (Ser)	0.38	Isoleucine (Iso)	0.56	Arginine (Arg)	0.60
Glutamic acid (Glu)	3.04	Leucine (Leu)	0.88	Proline (Pro)	0.54
Glycine (Gly)	0.45	Tyrosine (Tyr)	0.34		
Alanine (Ala)	1.10	Phenylalanine (Phe)	0.54		

SI: *Sarcodon imbricatus*

**Table 5:** The composition of minerals (including heavy metals) in SI.

Compounds	Contents (mg/100 g)	Compounds	Contents (mg/kg)
Kalium (K) (×10^2^)	39.57	Selenium (Se)	ND^①^
Natrium (Na)	14.52	Lead (Pb)	ND^②^
Calcium (Ca)	68.04	Mercury (Hg)	ND^③^
Cuprum (Cu)	3.31	Arsenic (As)	ND^④^
Ferrum (Fe)	78.42	Cadmium (Cd)	ND^⑤^
Zinc (Zn)	10.92	Chromium (Cr)	ND^⑥^
Manganese (Mn)	3.30		

SI: *Sarcodon imbricatus*; ND^①^: not detected (the detection limit was 2 mg/kg); ND^②^: not detected (the detection limit was 1 mg/kg); ND^③^: not detected (the detection limit was 0.1 mg/kg); ND^④^: not detected (the detection limit was 0.5 mg/kg); ND^⑤^: not detected (the detection limit was 0.5 mg/kg); ND^⑥^: not detected (the detection limit was 1 mg/kg).

**Table 6:** The effects of SI on oxidative stress-related factors in serum, liver, and muscle of acute excise-treated mice.

		CTRL	SI (g/kg)			GS (g/kg)
			0.25	0.5	1	0.05
Serum	MDA (nmol/mL)	8.4 ± 2.0	8.0 ± 1.4	7.9 ± 2.2	6.9 ± 1.3^#^	6.7 ± 1.7^#^
	ROS (U/mL)	461.0 ± 20.0	442.6 ± 21.9	401.6 ± 17.8^##^	415.4 ± 28.6^#^	381.9 ± 11.9^##^
	SOD (U/mL)	74.5 ± 12.0	83.6 ± 5.7	85.8 ± 11.0^#^	86.3 ± 13.0^#^	85.1 ± 8.8^#^
	GSH-Px (U/mL)	418.4 ± 90.8	518.2 ± 170.1^#^	539.2 ± 156.8^#^	559.2 ± 38.7^##^	568.8 ± 97.4^##^

Liver	MDA (nmol/mgprot)	5.2 ± 0.7	4.4 ± 0.3^#^	4.8 ± 0.3	3.7 ± 0.4^##^	4.6 ± 0.5^#^
	ROS (FI/gprot)	22103.5 ± 7400.4	25042.9 ± 9027.8	17296.6 ± 1974.0^#^	14914.6 ± 4638.8^##^	18780.5 ± 5160.0^#^
	SOD (U/mgprot)	234.9 ± 48.8	273.3 ± 49.8	340.7 ± 91.8^##^	256.3 ± 49.3	343.1 ± 49.4^##^
	GSH-Px (*μ*mol/gprot)	780.0 ± 195.0	818.3 ± 84.9	856.3 ± 19.4^#^	790.8 ± 85.1	886.2 ± 140.6^#^

Muscle	MDA (nmol/mgprot)	23.7 ± 4.6	24.2 ± 2.2	22.7 ± 2.2	21.6 ± 2.7	22.3 ± 2.1
	ROS (FI/gprot)	61278.3 ± 8914.2	59534.4 ± 4636.2	53426.1 ± 333.9^#^	51847.6 ± 5748.2^#^	47689.1 ± 6061.2^##^
	SOD (U/mgprot)	115.5 ± 23.4	122.4 ± 30.3	187.3 ± 24.6^##^	179.6 ± 19.4^#^	204.5 ± 29.7^##^
	GSH-Px (*μ*mol/gprot)	653.9 ± 75.4	663.3 ± 43.1	702.4 ± 32.8	660.9 ± 46.8	756.2 ± 65.9^#^

Treatment with SI (0.25 g/kg, 0.5 g/kg, and 1.0 g/kg) and GS (0.05 g/kg) for 18 days; after a 30 min swimming, the levels of MDA and ROS and the activities of SOD and GSH-Px in serum, liver, and muscle were detected. The data were analyzed using a one-way ANOVA followed by Dunn's test and expressed as means ± SD (*n* = 10/group). ^#^*P* < 0.05 and ^##^*P* < 0.01 versus the control group. SI: *S. imbricatus*; GS: *Ginsenoside.*

**Table 7:** The effects of SI on oxidative stress-related factors in serum, liver, and muscle of CFS mice.

		CTRL	Model	SI (g/kg)			GS (g/kg)
				0.25	0.5	1	0.05
Serum	MDA (nmol/mL)	22.4 ± 2.6	28.2 ± 1.5^#^	21.8 ± 3.0	20.2 ± 1.4^∗∗^	22.1 ± 0.9	19.9 ± 1.2^∗∗^
	ROS (U/mL)	203.3 ± 19.4	263.1 ± 9.0^#^	237.3 ± 15.1	232.5 ± 21.2^∗^	221.7 ± 16.8^∗^	224.4 ± 23.5^∗^
	SOD (U/mL)	158.2 ± 11.2	129.7 ± 7.6^#^	137.6 ± 8.7	139.7 ± 11.9	154.8 ± 23.6^∗^	142.2 ± 21.1^∗^
	GSH-Px (U/mL)	230.4 ± 29.8	192.0 ± 15.5^#^	209.2 ± 27.0	203.5 ± 19.8	218.7 ± 21.2^∗^	220.8 ± 10.6^∗^

Liver	MDA (nmol/mgprot)	4.5 ± 0.5	5.6 ± 0.7^#^	5.3 ± 1.3	5.5 ± 0.7	4.5 ± 0.9^∗^	4.5 ± 0.6^∗^
	ROS (U/mgprot)	384.8 ± 41.6	449.2 ± 9.4^#^	420.3 ± 29.6	372.2 ± 39.8^∗^	379.1 ± 29.7^∗^	358.7 ± 20.2^∗∗^
	SOD (U/mgprot)	136.1 ± 12.2	105.4 ± 8.2^##^	125.3 ± 25.1	128.5 ± 24.5^∗^	120.4 ± 30.2	136.9 ± 23.9^∗^
	GSH-Px (U/mgprot)	172.7 ± 13.6	110.5 ± 13.2^##^	163.9 ± 23.5^∗^	169.5 ± 24.5^∗^	157.6 ± 16.8	168.2 ± 27.4^∗^

Muscle	MDA (nmol/mgprot)	6.1 ± 1.2	7.5 ± 1.1^#^	6.7 ± 1.2	6.3 ± 1.4	6.1 ± 0.7	6.0 ± 0.8^∗^
	ROS (U/mgprot)	336.5 ± 39.8	402.2 ± 26.8^##^	423.2 ± 34.2	358.0 ± 37.9^∗∗^	369.7 ± 38.0^∗^	349.6 ± 36.2^∗∗^
	SOD (U/mgprot)	289.6 ± 48.6	210.2 ± 19.8^#^	277.7 ± 35.4	297.2 ± 66.7^∗^	311.1 ± 29.7^∗∗^	287.9 ± 42.9^∗^
	GSH-Px (U/mgprot)	167.7 ± 61.7	150.6 ± 30.7^#^	160.0 ± 47.1	170.5 ± 33.2^∗^	165.2 ± 48.1	174.0 ± 47.3^∗^

Treatment with SI (0.25 g/kg, 0.5 g/kg, and 1.0 g/kg) and GS (0.05 g/kg) for 32 days in CFS mice; the levels of MDA and ROS and the activities of SOD and GSH-Px in serum, liver, and muscle were detected. The data were analyzed using a one-way ANOVA followed by Dunn's test and expressed as means ± SD (*n* = 10/group). ^#^*P* < 0.05 and ^##^*P* < 0.01 versus the control group; ^∗^*P* < 0.05 and ^∗∗^*P* < 0.01 versus the model group. SI: *S. imbricatus*; GS *Ginsenoside.*

## Abbreviations

Ala: Alanine
ANOVA: Analysis of variance
ARE: Antioxidant responsive element
Asp: Aspartic acid
ATP: Adenosine triphosphate
BCA: Bicinchoninic acid
BSA: Bovine serum albumin
BUN: Blood urea nitrogen
CAT: Catalase
CFS: Chronic fatigue syndrome
CTX: Cyclophosphamide
CMC-Na: Sodium carboxymethyl cellulose
ELISA: Enzyme-linked immunosorbent assay
FST: Forced swimming test
GAPDH: Glyceraldehyde 3-phosphate dehydrogenase
Glu: Glutamic acid
GS: Ginsenoside
GSH: Glutathione
GSH-Px: Glutathione peroxidase
HO-1: Heme oxygenase-1
RRT: Rota-rod test
LD: Lactic acid
MDA: Malondialdehyde
Nrf2: Nuclear factor-erythroid 2-related factor 2
ROS: Reactive oxygen species
SD: Standard deviation
SOD: Superoxide dismutase
TST: Tail suspension test.
